# Survival Outcomes in Patients With 2018 FIGO Stage IA2–IIA2 Cervical Cancer Treated With Laparoscopic *Versus* Open Radical Hysterectomy: A Propensity Score-Weighting Analysis

**DOI:** 10.3389/fonc.2021.682849

**Published:** 2021-06-17

**Authors:** Wancheng Zhao, Yunyun Xiao, Wei Zhao, Qing Yang, Fangfang Bi

**Affiliations:** ^1^ Department of Obstetrics and Gynecology, Shengjing Hospital of China Medical University, Shenyang, China; ^2^ Department of Gynecology and Obstetrics, Dalian Obstetrics and Gynecology Hospital Affiliated to Dalian Medical University & Dalian Maternal and Child Health Care Hospital, Dalian, China

**Keywords:** laparoscopy, open, radical hysterectomy, cervical cancer, Federation International of Gynecology and Obstetrics, National Comprehensive Cancer Network

## Abstract

**Objective:**

To compare the survival and recurrence outcomes between open and laparoscopic radically hysterectomy (RH) for stage IA2-IIA2 cervical cancer based on Federation International of Gynecology and Obstetrics (FIGO) 2018.

**Methods:**

Data of 1,373 early cervical cancer patients undergoing open or laparoscopic radically hysterectomy at ShengJing Hospital of China Medical University between January 1, 2013, and December 31, 2016, were retrospectively reviewed. Propensity score-based inverse probability of treatment weighting (PS-IPTW) was used to balance the covariates between the two groups.

**Results:**

A total of 705 cervical cancer patients of FIGO 2009 stage IA2-IIA2 were finally enrolled in this study. After IPTW adjustment, the OS (HR = 2.095, 95% CI: 1.233-3.562, P = 0.006) and PFS (HR = 1.950, 95%CI: 1.194-3.184, P = 0.008) rates were significantly higher in the open RH (ORH) group compared with the laparoscopic RH (LRH) group. Then after re-staging according to the FIGO 2018 staging system, 561 patients still belonged to stage IA2-IIA2, 144 patients were upgraded to stage IIIC1p-IIIC2p. The ORH group had a significantly superior OS (HR = 1.977, 95%CI: 1.077-3.626, P = 0.028) and PFS (HR = 1.811, 95%CI: 1.046-3.134, P = 0.034) compared with the LRH group after PS-IPTW analysis. Furthermore, in patients with no high and intermediate risks, difference of the OS (HR = 1.386, 95%CI: 0.287-6.69, P = 0.684) and PFS (HR = 1.524, 95%CI: 0.363-6.396, P = 0.565) rates between the two groups were with no statistical meaning.

**Conclusions:**

Outcomes of this retrospective cohort study were in compliance with indications for ORH recommended by the National Comprehensive Cancer Network guidelines Version 1, 2021. However, LRH showed non-inferiority for patients with no prognostic risk factors compared with ORH.

## Introduction

Cervical cancer was the fourth most common cancer in women worldwide. The estimated global yearly incidence of cervical cancer in 2018 was 570,000 cases, among which China contributing approximately one fifth (1). Guidelines indicate that radical hysterectomy is standard treatment for early-stage cervical cancer. However, disputes about the prognostic outcomes of the cervical cancer patients underwent the open radically hysterectomy (ORH) or minimally invasive surgery (MIS) laparoscopic hysterectomy had persisted nearly 25 years since the introduction of the laparoscopic approach in 1992 (2–18). Unexpectedly, a phase III randomized, open-label, non-inferiority clinical trial named the Laparoscopic Approach to Cervical Cancer identified that cervical cancer patients of Federation International of Gynecology and Obstetrics (FIGO) 2009 stage IA1 with lymphovascular invasion, IA2 and IB2 in the MIS group had almost four times the risk of recurrence and 6 times the risk of death compared with the women in the ORH group (7). Afterward, a meta-analysis, which included 15 high-quality observational studies comprising 9,499 cervical cancer patients of FIGO 2009 stage IA1-IIA2 (stage IA1 with lymphovascular invasion), concluded that patients in the ORH group had superior overall and disease-free survival than patients in the MIS group (6). Besides, the European Society of Gynecological Oncology (ESGO), the British Gynecological Cancer Society (BGCS), and two other epidemiologic studies also had the same opinion (9, 19, 20).

Based on the most recent findings, the National Comprehensive Cancer Network (NCCN) Guidelines Version 1.2021 recommended that ORH was the primary treatment for FIGO 2018 stage IA2, IB1, IB2 and IIA1 cervical cancer patients. As for operable cervical cancer patients with FIGO 2018 stage IB3, IIA2, and IIICr, laparoscopic approach is absolutely prohibited. The panel had updated the guidelines according to the revised 2018 FIGO staging system. However, trial data utilized in the guidelines were all from the previous 2009 FIGO staging system.

Accordingly, this study is conducted to compare the survival and recurrence outcomes between ORH and laparoscopic radically hysterectomy (LRH) for stage IA2-IIA2 cervical cancer patients comprising FIGO 2018 staging system. In addition, three subgroups of FIGO 2018 stage IA1, IB1, IB2 and IIA1, FIGO 2018 stage IB3 and IIA2, FIGO 2018 stage IIIC1p-IIIC2p referring to the NCCN guidelines Version 1.2021 were analyzed between the two surgical approaches. As squamous cell carcinomas account for approximately 75% to 80% of all cervical cancers (21), and stromal invasion pattern was considered to play better roles on predicting the prognosis of adenocarcinoma and adenosquamous carcinoma than FIGO stage system (22), the histology type of patients included in this study is only squamous cell carcinoma.

## Methods

### Inclusion and Exclusion Criteria

**Figure 1:**
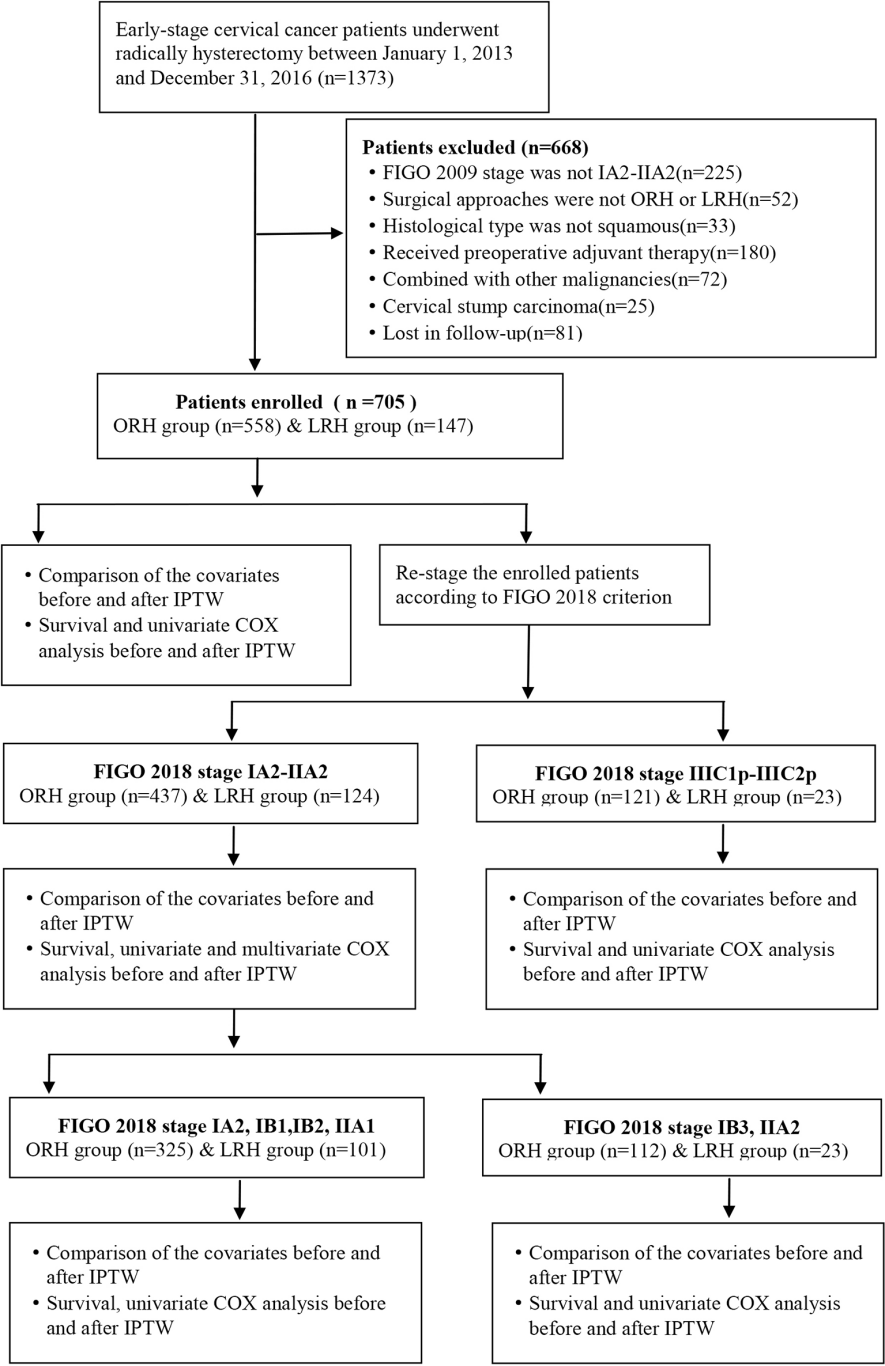
Workflow of patient inclusion and statistical analysis.

This is a retrospective, single-center study of cervical cancer patients undergoing surgery at ShengJing Hospital of China Medical University between January 1, 2013, and January 1, 2016. The inclusion criteria were as follows: (1) the clinical diagnosis of the participants was International Federation of Gynecology and Obstetrics (FIGO) 2009 stage IA2-IIA2; (2) surgery could be accomplished *via* either open or laparoscopic radically hysterectomy and pelvic lymphadenectomy, with or without para-abdominal aortic lymphadenectomy; (3) the histological diagnosis was squamous cell carcinoma; (4) no neoadjuvant radiation or chemotherapy prior to surgery. The exclusion criteria were as follows: (1) pregnancy combined with cervical cancer; (2) combined with other malignant or borderline tumors at other sites; (3) cervical stump cancer. And the study was approved by the ethics review board of ShengJing Hospital of China Medical University. Inclusion condition of the participants is shown in **Figure 1**.

### Surgical Approach and Observation Index

The radical hysterectomy includes resection of the uterus as far as possible from the uterosacral ligament, excision of the parametrial tissue as near as to the pelvic wall and removal of the upper 1/3 of the vagina. Extent of radical hysterectomy was evaluated according to Querleu and Morrow classification (23). The following data were collected from the medical records of the included participants: demographic information, body mass index (BMI), operation year, histological type, clinical stage (FIGO 2009), tumor size (pathologic tumor size), tumor grade, pathological results, and adjuvant treatment condition. And all the patients were re-staged according to the FIGO 2018 criterion.

### Follow-Up

After surgery, patients were informed to come back for checkup every 3 to 6 months for the first 2 years, 6 to 12 months for 3 to 5 years, then annually thereafter. The follow-up procedures were conducted by professional gynecologists through telephone. And the last follow-up time was December 31, 2020. The postoperative adjuvant treatments, survival status, time of death, reasons for death, recurrence time, and location were recorded. The primary endpoint was overall survival (OS) which was defined as the time (months) from initial diagnosis to death from any causes. The secondary endpoint was progression-free survival (PFS) which was defined as the time (months) from diagnosis to disease recurrence. Data of patients with no evidence of death or recurrence were censored.

### Statistical Analysis

Continuous quantitative data are presented as mean ± standard deviation (SD) and analyzed through Student t test. Categorical data are presented as numbers and percentages, and analyzed through Chi-square (χ^2^) or Fisher exact test for non-ordinal variables, Mann-Whitney U test for ordinal variables (24). Survival analysis was conducted through Kaplan-Meier method and compared with log-rank test (25). Univariate and multivariate Cox proportional hazards regression analyses were also applied to calculate the hazard ratio (HR) and 95% confidence internal (CI) associated with the recurrence and survival outcomes of the cervical cancer patients (26).

Propensity score-based inverse probability of treatment weighting (PS-IPTW) was used to balance the covariates associated with the recurrence and survival outcomes of the cervical cancer patients between the ORH and LRH groups. And the covariates include: age, operation year, clinical stage, tumor size, tumor grade, stromal invasion, lymphovascular space invasion (LVSI), pelvic node, aortic node, parametrial invasion, vaginal margin invasion, nerve invasion, and chemoradiotherapy condition. The IPTW approach assigned in LRH group was weight = Pt/PS, and weight = (1 − Pt)/(1 − PS) in the ORH group (27). Pt was the percentage of the number of the LRH group according to the total patients. PS was the propensity score of each patient. The data analysis was conducted with Statistical Package for Social Sciences (IBM SPSS, Version 25, Armonk, NY) and R version 4.0.3 (R Foundation for Statistical Computing, Vienna, Austria). A p-value < 0.05 indicated a statistically significant difference.

## Results

### Patient Enrollment

A total of 1,373 early-stage cervical cancer patients were identified accepting operation in ShengJing Hospital of China Medical University between January 1, 2013, and January 1, 2016. As shown in **Figure 1**, through absolute inclusion and exclusion criteria, 705 cervical cancer patients of FIGO 2009 stage IA2-IIA2 were finally enrolled in this study, with 558 patients in the ORH group and 147 patients in the LRH group. Then all the enrolled patients were re-staged according to the FIGO 2018 classification. Distribution of patients’ clinical stages according to FIGO 2009 and FIGO 2018 criteria correspondingly was exhibited in **Supplementary Table 1**. Accordingly, 561 patients still belonged to stage IA2-IIA2, with 437 patients in the ORH group and 124 patients in the LRH group. And 144 patients were upgraded to FIGO 2018 stage IIIC1p-IIIC2p, with 121 patients in the ORH group and 23 patients in the LRH group.

### Characteristics of the Covariates

Comparison of the clinicopathological parameters of stage IA2-IIA2 before and after IPTW adjustment between the ORH and the LRH group on the basis of FIGO 2009 and FIGO 2018 criteria were listed in **Tables 1** and **2**, respectively. In the original sample, age at operation, operation year, clinical stage, tumor size, and stromal invasion condition were obviously discrepant between the two groups (P<0.05). After IPTW adjustment, no difference of the covariates was identified between the two groups (P>0.05). As the data of BMI were not recorded adequately, which were not included in the covariates. Similarly, the covariates between the two groups in the three subgroups were also balanced with IPTW analysis.

**Table 1:** Patient demographics and tumor characteristics between open and laparoscopic radically hysterectomy of stage IA2-IIA2 based on FIGO 2009 before and after propensity score weighting.

Variables	Original sample			IPTW sample		
	Open (n=558)	Laparoscopy (n=147)	P	Open (n=558 )	Laparoscopy (n=143)	P
Age at Operation	49.81±9.480	47.36 ± 8.698	0.005^a^	49.31±9.564	48.74±8.497	0.486
Operation Year			0.000^a^			0.379
2013	57 (10.2)	3 (2)		50 (9)	4 (2.8)	
2014	94 (16.8)	17 (11.6)		86 (15.4)	24 (16.8)	
2015	224 (40.2)	59 (40.1)		222 (39.9)	69 (48.3)	
2016	183 (32.8)	68 (46.3)		199 (35.7)	46 (32.2)	
Clinical Stage			0.000^a^			0.110
IA2	10 (1.8)	3 (2)		13 (2.3)	2 (1.4)	
IB1	265 (47.5)	107 (72.8)		285 (51.1)	84 (58.7)	
IB2	64 (11.5)	7 (4.8)		63 (11.3)	12 (8.4)	
IIA1	133 (23.8)	18 (12.2)		123 (22.0)	22 (15.4)	
IIA2	84 (15.4)	12 (8.2)		74 (13.3)	23 (16.1)	
Tumor Grade			0.232			0.657
High	73 (13.1)	23 (15.6)		77 (13.8)	20 (14.1)	
Middle	458 (82.1)	120 (81.6)		455 (81.5)	115 (81)	
Low	27 (4.8)	4 (2.8)		26 (4.7)	7 (4.9)	
Tumor size			0.001^a^			0.670
<2 cm	83 (14.9)	39 (26.5)		95 (17.1)	32 (25.8)	
2–4 cm	321 (57.5)	80 (54.4)		320 (57.5)	69 (79)	
≥4 cm	154 (27.9)	27 (18.4)		142 (25.5)	41 (37.2)	
Stromal Invasion			0.000^a^			0.336
≤1/2	136 (24.4)	58 (39.5)		156 (28)	44 (31)	
>1/2	422 (75.6)	89 (60.5)		402 (72)	98 (9)	
LVSI	219 (39.2)	45 (30.6)	0.054	211 (37.8)	59 (41.5)	0.414
Parametrial Invasion	6 (1)	1 (0.7)	0.553^b^	6 (1.1)	1 (0.7)	0.568^b^
Vaginal Margin	9 (1.6)	1 (0.7)	0.349^b^	8 (1.4)	2 (1.4)	0.670^b^
Positive Nodes						
Pelvic	120 (21.5)	23 (15.6)	0.116	113 (20.3)	26 (18.3)	0.605
Para aortic	5 (0.9)	1 (0.7)	0.634^b^	5 (0.9)	1 (0.7)	0.646^b^
Nerve invasion	36 (6.5)	10 (6.8)	0.878	38 (6.8)	13 (9.2)	0.337
Adjuvant Therapy						
Chemotherapy(≥1 dose)	302 (54.1)	74 (50.3)	0.414	297 (53.2)	77 (53.8)	0.894
Radiotherapy(≥1 dose)	347 (62.2)	96 (65.3)	0.486	349 (62.5)	90 (63.4)	0.854

FIGO, Federation International of Gynecology and Obstetrics; IPTW, Inverse Probability of Treatment Weighting; LVSI, lymphovascular space invasion;

^a^P<0.05; ^b^Fisher exact examination.

**Table 2:** Patient demographics and tumor characteristics between open and laparoscopic radically hysterectomy of stage IA2-IIA2 based on FIGO 2018 before and after propensity score weighting.

Variables	Original sample			IPTW sample		
	Open (n=437)	Laparoscopy (n=124)	P	Open (n=437 )	Laparoscopy (n=119)	P
Age at operation	50.42±9.358	47.66±8.736	0.003^a^	49.81±9.513	49.2±8.351	0.498
Operation year			0.001^a^			0.092
2013	45 (10.3)	2 (1.6)		39 (8.9)	3 (2.5)	
2014	65 (14.9)	13(10.5)		58(13.3)	20 (16.8)	
2015	184 (42.1)	49 (39.5)		182 (41.6)	55 (46.2)	
2016	143 (32.7)	60 (48.5)		158 (36.2)	41 (34.5)	
Clinical stage			0.000^a^			0.329
IA2	10 (2.3)	2 (2.4)		12 (2.7)	2 (1.7)	
IB1	53 (12.1)	29(23.4)		64 (14.6)	21 (17.6)	
IB2	169 (38.7)	54 (43.5)		169 (38.7)	48 (40.3)	
IB3	50 (11.4)	13 (10.5)		50 (11.4)	16(13.4)	
IIA1	93 (21.3)	15(12.1)		87 (19.9)	17 (14.3)	
IIA2	62 (1.2)	10 (8.1)		55 (12.6)	15 (12.6)	
Tumor grade			0.606			0.792
High	57 (13.0)	17 (13.7)		59 (13.5)	16 (13.3)	
Middle	359 (82.2)	103 (83.1)		358 (81.7)	98 (81.7)	
Low	21 (4.8)	4 (3.2)		21 (4.8)	6 (5.0)	
Tumor size			0.008^a^			0.652
<2 cm	75 (17.2)	35 (28.2)		87 (20)	29 (24.4)	
2–4 cm	251 (57.4)	66 (53.2)		245 (56.2)	59 (49.6)	
≥4 cm	111 (25.4)	23 (18.5)		104 (23.9)	31 (26.1)	
Stromal invasion			0.005^a^			0.484
≤1/2	131 (30.0)	54 (43.5)		146 (33.4)	44 (37)	
>1/2	306 (17.0)	70 (56.5)		291 (66.6)	75 (63)	
LVSI	150 (34.3)	34 (27.4)	0.148	146 (33.4)	46 (38.7)	0.286
Parametrial invasion	3 (0.7)	0 (0)	0.472^b^	2 (0.5)	0 (0)	0.617^b^
Vaginal margin	7 (1.6)	0 (0)	0.172^b^	5 (1.1)	0 (0)	0.298^b^
Nerve invasion	23 (5.3)	8 (6.5)	0.609	25 (5.7)	7 (7.6)	0.457
Adjuvant therapy						
Chemotherapy(≥1 dose)	209 (47.8)	51 (41.1)	0.187	202 (46.2)	53 (44.5)	0.743
Radiotherapy(≥1 dose)	245 (56.1)	74 (59.7)	0.473	248 (56.8)	69 (58.0)	0.810

FIGO, Federation International of Gynecology and Obstetrics; IPTW, Inverse Probability of Treatment Weighting; LVSI, lymphovascular space invasion.

^a^P < 0.05; ^b^Fisher exact examination.

### Survival Analysis

**Figure 2:**
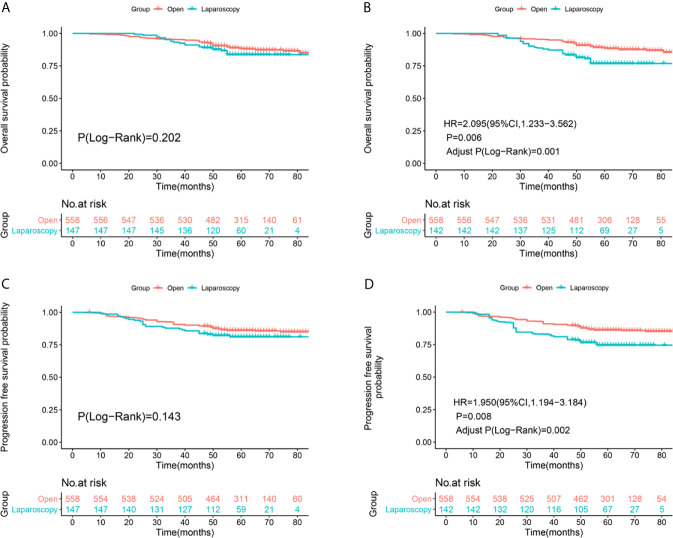
Survival and recurrence outcomes between open and laparoscopic radically hysterectomy for stage IA2-IIA2 cervical cancer patients based on Federation International of Gynecology and Obstetrics (FIGO) 2009 staging system. **(A)** Overall survival (OS) curves of the patients before propensity score-based inverse probability of treatment weighting (PS-IPTW) analysis. **(B)** OS curves of the patients after IPTW analysis. **(C)** Progression free survival (PFS) curves of the patients before IPTW analysis. **(D)** PFS curves of the patients after IPTW analysis.

Median follow-up was 61 months (range, 23–94 months) in the ORH group *versus* 57.5 months (range, 26–88 months) in the LRH group. And among all the enrolled patients, 66 patients died and 78 patients had recurrence in the ORH group, 22 patients died and 27 patients had recurrence in the LRH group up to December 31, 2020. The 5-year OS rates were 88.4% in the ORH group and 83.7% in the LRH group (Log-Rank P=0.202, **Figure 2A**), respectively. The 5-year PFS rates were 86% in the ORH group and 81.2% in the LRH group (Log-Rank P=0.143, **Figure 2C**), respectively. After IPTW adjustment, the OS (HR = 2.095, 95% CI: 1.233-3.562, P = 0.006, Adjust Log-Rank P= 0.001, **Figure 2B**) and PFS (HR=1.950, 95%CI: 1.194-3.184, P=0.008, Adjust Log-Rank P= 0.002, **Figure 2D**) rates were significantly higher in the ORH group compared with the LRH group.

**Figure 3:**
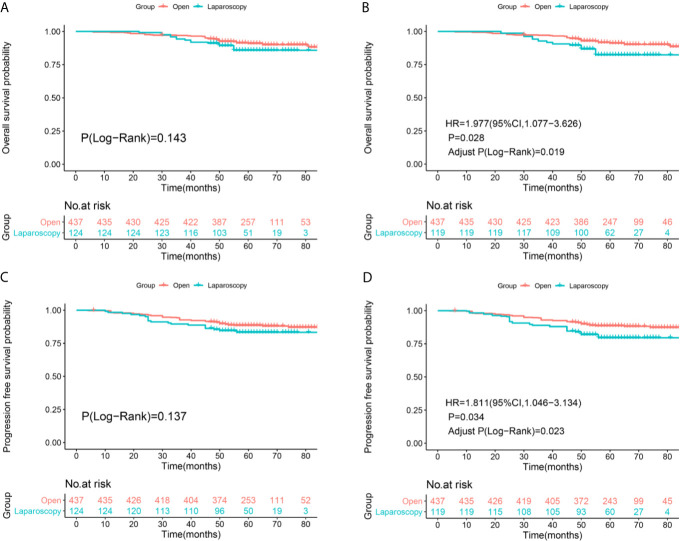
Survival and recurrence outcomes between open and laparoscopic radically hysterectomy for stage IA2-IIA2 cervical cancer patients based on Federation International of Gynecology and Obstetrics (FIGO) 2018 staging system. **(A)** Overall survival (OS) curves of the patients before propensity score-based inverse probability of treatment weighting (PS-IPTW) analysis. **(B)** OS curves of the patients after IPTW analysis. **(C)** Progression free survival (PFS) curves of the patients before IPTW analysis. **(D)** PFS curves of the patients after IPTW analysis.

After re-staging according to FIGO 2018 staging system, non-inferiority of the OS (Log-Rank P=0.143, **Figure 3A**) and PFS (Log-Rank P=0.137, **Figure 2C**) rates were identified between the two surgical approaches of stage IA2-IIA2 before IPTW adjustment (**Figures 3A, C** **)**. There were 40 deaths in the ORH group and 16 deaths in the LRH group, with 5-year OS rates of 91.1% and 85.8%, respectively. There were 51 recurrences in the ORH group and 20 recurrences in the LRH group, with 5-year PFS rates of 88.6% and 83.3% separately. However, after PS-IPTW analysis, the ORH group had a significantly superior OS (HR=1.977, 95%CI: 1.077-3.626, P=0.028, Adjust Log-Rank P= 0.019, **Figure 3B**) and PFS (HR=1.811, 95%CI: 1.046-3.134, P=0.034, Adjust Log-Rank P= 0.023, **Figure 3D**) compared with the LRH group.

At the same time, three subgroup analyses were also conducted in this study (**Supplementary Figures 1**-**4**). In patients of FIGO 2018 stage IIIC1p-IIIC2p, 26 patients died in the ORH group and six patients died in the LRH group, with 5-year OS rates of 78.6% and 72.2%, respectively (Log-Rank P=0.579). Besides, 27 patients had recurrence in the ORH group, and seven patients had recurrence in the LRH group, with 5-year PFS rates of 77% and 69.6%, respectively (Log-Rank P = 0.387). Through PS-IPTW, the OS (HR=1.869, 95%CI: 0.720-4.851, P=0.199, Adjust Log-Rank P= 0.212) and PFS (HR=1.004, 95%CI: 0.761-4.755, P=0.169, Adjust Log-Rank P= 0.191) rates showed no obvious difference between the two groups (**Supplementary Figure 1**).

In patients of FIGO 2018 stage IA2, IB1, IB2, and IIA1, 28 patients died in the ORH group and 6 patients died in the LRH group, with 5-year OS rates of 91.6% and 93.5%, respectively (Log-Rank P=0.507). Besides, 32 patients had recurrence in the ORH group and nine patients had recurrence in the LRH group, with 5-year PFS rates of 90.6% and 90.5% (Log-Rank P=0.908). After IPTW analysis, non-inferiority of the OS (HR = 0.784, 95% CI: 0.312-1.966, P=0.604, Adjust Log-Rank P= 0.574) and PFS (HR=1.069, 95%CI: 0.466-2.453, P=0.875, Adjust Log-Rank P= 0.856) rates was identified between the two surgical approaches (**Supplementary Figure 2**). Furthermore, in patients with no high and intermediate risks, six patients died in the ORH group and 2 patients in the LRH group, with 5-year OS rate of 94.2% and 91.6%, respectively (Log-Rank P=0.778). Then six patients had recurrence in the ORH group, and three patients had recurrence in the LRH group, with 5-year PFS rates of 92.3% *vs* 91.7%, respectively (Log-Rank P=0.906). After PS-IPTW, difference of the OS (HR=1.386, 95%CI: 0.287-6.69, P=0.684, Adjust Log-Rank P= 0.498) and PFS (HR=1.524, 95%CI: 0.363-6.396, P=0.565, Adjust Log-Rank P=0.612) rates between the two groups were still with no statistical meaning (**Supplementary Figure 3**).

In the subgroup of FIGO 2018 stage IB3 and IIA2, 12 patients died and 19 patients had recurrence in the ORH group, 10 patients died and 11 patients had recurrence in the LRH group. The 5-year OS rates were 89.4% in the ORH group and 53.4% in the LRH group (Log-Rank P<0.001). The 5-year PFS rates were 82.2% in the ORH group and 52.2% in the LRH group (Log-Rank P<0.001). After IPTW adjustment, the OS (HR=3.498, 95%CI: 0.902-13.57, P=0.070, Adjust Log-Rank P= 0.005) and PFS (HR=2.369, 95%CI: 0.642-8.741, P=0.195, Adjust Log-Rank P= 0.034) rates were superior in the ORH group compared with the LRH group (**Supplementary Figure 4**).

### Analysis of Survival Factors

**Figure 4:**
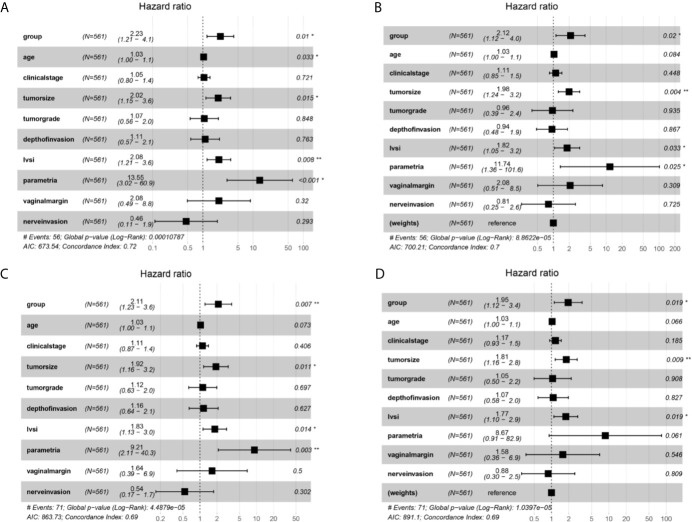
Multivariate Cox proportional hazards regression analysis outcomes of the survival factors associated with overall survival (OS) and progression free survival (PFS) for patients in stage IA2-IIA2 cervical cancer based on Federation International of Gynecology and Obstetrics (FIGO) 2018 staging system. **(A)** Overall survival (OS) curves of the patients before propensity score-based inverse probability of treatment weighting (PS-IPTW) analysis. **(B)** OS curves of the patients after IPTW analysis. **(C)** Progression free survival (PFS) curves of the patients before IPTW analysis. **(D)** PFS curves of the patients after IPTW analysis. *P<0.05, **P<0.01.

Multivariate Cox analysis was further applied to identify the survival factors associated with the PFS and OS of FIGO 2018 stage IA2-IIA2 patients. As exhibited in **Figure 4A**, before adjustment, group, age, tumor size, LVSI, and parametrial invasion were proven to be associated with the survival condition of the cervical cancer patients. Then group, tumor size, LVSI, and parametrial invasion were identified to be connected with the recurrence of patients before adjustment (**Figure 4C**). After IPTW adjustment, group, tumor size, LVSI were confirmed to be significantly associated PFS and OS of cervical cancer patients of FIGO stage IA2-IIA2 consistently (**Figures 4B, D** **)**. Besides, age and parametrial invasion also showed critically significant P-value.

## Discussion

Since Nezhat reported the first case of LRH in 1992 (2), numerous retrospective studies and meta-analysis had proven that LRH had less bleeding, lower risk of infection, more rapid postoperative recovery, and shorter hospital stay compared with ORH (3, 5, 8). Moreover, LRH had not shown inferior 5-year overall or disease-free survival rates than ORH (5, 8, 28–33). Therefore, LRH for cervical cancer had been gradually accepted and popularized by both doctors and patients. The 2020 NCCN Guidelines still recommended treating stage IA2-IIA cervical cancer with ORH, LRH or robot-assisted LRH. However, trends had changed since the publication of the LACC trial (7). MIS was only recommended for extrafascial hysterectomy and fertility-sparing radically trachelectomy for early cervical cancer in the NCCN guidelines Version 1.2021. In addition, the FIGO Committee for Gynecologic Oncology revised the staging system of cervical cancer in 2018 (34), allowing the use of any imaging modality and/or pathological findings for allocating the stage. For the early-stage cervical cancer, the lateral extent of the lesion was not considered in stage IA, an additional cutoff at 2.0 cm was introduced in stage IB, and any patient with positive lymph nodes was upgraded to stage IIIC in the new criterion.

This study was conducted to compare the survival outcomes of the ORH and LRH comprising the new FIGO stage system. First, we identified that LRH was associated with worse 5-year overall (83.7% *vs* 88.4%) and progression-free (81.2% *vs* 88.6%) survival outcomes compared with ORH in FIGO 2009 stage IA2-IIA2 cervical cancer patients. It is consistent with the modern opinion about LRH. Second, patients of FIGO 2018 stage IA2-IIA2 in the ORH group also have superior 5-year OS (91.1% *vs* 85.8%) and 5-year PFS (88.6% *vs* 83.3%) rates than the LRH group. Third, in the subgroup analysis for patients of FIGO 2018 stage IA2, IB1-IB2, and IIA1, the LRH group showed non-inferior 5-year OS (93.5% *vs* 91.6%) and 5-year PFS (90.5% *vs* 90.6%) comparing to the ORH group. Fourth, in the subgroup of FIGO 2018 stage IB3 and IIA2, patients underwent LRH had obviously poor 5-year OS (53.4% *vs* 89.4%) and 5-year PFS (52.2% *vs* 82.2%) compared with patients underwent ORH. Patients in the LRH group almost had three times risk of death and 2 times risk of recurrence than the ORH group. Last, in the subgroup of FIGO 2018 stage IIIC1p-IIIC2p, though patients undergoing LRH suffered from obviously poor 5-year OS (72.2% *vs* 78.6%) and 5-year PFS (69.6% *vs* 77%) than patients undergoing ORH, which was not statistically significant.

In general, the survival outcomes in our study were in accordance with several population-based and high-volume institutional retrospective studies, confirming the opinion of LACC trial, which concluded that MIS increased recurrence and death for early cervical cancer patients, as the **Table 2** in the research of Yang et al. has shown (35). At the same time, multivariate Cox analysis was also conducted to discover the prognostic factors associated with the recurrence and survival outcomes of cervical cancer with FIGO 2018 stage IA2-IIA2. Surgical approach, tumor size, LVSI, and parametrial invasion were found to be independent prognostic factors. The finding was essentially in agreement with the pathologic risk factors of cervical cancer. Besides, patients in the LRH group were proven to have almost two times of death and recurrence than the ORH group, which further supported the conclusion of the LACC Trial (7).

Overall, results of our study supported the indications of ORH for early-stage cervical cancer patients based on the FIGO 2018 staging system, which was in compliance with the NCCN guidelines Version 1.2021. However, in patients of FIGO 2018 stage IA2, IB1-IB2, and IIA1, LRH showed non-inferiority compared with ORH even after IPTW adjustment. This finding reminded us that the advantages of laparoscopic surgery in radical hysterectomy of cervical cancer could not be completely denied. Patients in this subgroup all had no “high-risk” prognostic factors, presenting negative nodes, negative margins, and negative parametria. According to the “intermediate-risk” Sedlis Criteria: 1) greater than 1/3 stromal invasion; 2) LVSI; or 3) cervical tumor diameters more than 4 cm (36, 37), several patients showed deep stromal invasion and positive LVSI. So LRH might be applicable for some specific cervical cancer patients. In the LACC trial, it emphasized that the results cannot be generalized to patients with “low-risk” cervical cancer (tumor size < 2 cm; no lymphovascular invasion; depth of invasion < 10 mm; and no lymph-node involvement) (7). Many previous studies also identified that LRH was non-inferior to ORH for early cervical cancer (5, 8, 28–33), especially in patients with conization before surgery and no visible tumor on the final pathology (38). In view of the currently negative point to LRH, we supposed that LRH might be applicable for early-stage cervical cancer patients without high and intermediate risk factors, including negative nodes, negative margins, negative parametria, less than 1/3 stromal invasion, negative LVSI, and tumor size less than 4 cm. And the data from our center also supposed this standpoint. Furthermore, as some studies have shown that patients with tumor size <2 cm have better prognosis, the tumor size could be set as < 2 cm as selection criterion more carefully. On this basis, patients should receive conization before LRH during clinical work (18, 39–43). Besides, histology should also be taken into consideration (22, 44, 45). Patients of FIGO stage IIIC1-IIIC2 in the LRH group have obviously worse PFS and OS than the ORH group, but which was not statistically significant. The reasons may be as follows: first, number of patients in the subgroup was limited, especially in the LRH group; second, though lymph node metastasis is an important factor associated with the prognosis of cervical cancer patients, stratifying the clinical stage according to the lymph node status individually is still controversial (46). If the local extent of the disease between the groups was not comparable, outcomes of the surgery might also be affected. However, individualized chemoradiotherapy is recommended for the FIGO 2018 IIIC1p-IIIC2p in the NCCN guidelines Version 1.2021. So the rationality of the clinical staging is better worth discussing than the operation way.

Several causes were proposed to explain the high risk of recurrence and poor survival in patients undergoing LRH, including the establishment of pneumoperitoneum through CO_2_ insufflation, application of uterine manipulators and the method of colpotomy. The continuously perfusing and flowing CO_2_ in the abdominopelvic cavity could lead to spread of the detached tumor cells (47–49). The compression effects of the uterine manipulator on the upper vagina might increase the risk of tumor cell detachment, especially bringing about the distant dissemination and metastasis of intra-luminal tumors in patients with positive LVSI (50, 51). The way of colpotomy was also considered to increase likely exposure of the tumor to the abdominal cavity at the end of the surgery (52, 53). These three reasons might also explain the results of our research. Besides, several studies identified that the most effective way to reduce the recurrence rate during the LRH is to avoid tumor dissemination, especially during the vaginal colpotomy (54–57). Kanao et al. have identified that the no-look no-touch technique may be useful to reduce recurrence risks through preventing intraoperative tumor spillage during LRH for early cervical cancer patients. The technique incorporates four specific measures: 1) creation of a vaginal cuff, 2) avoidance of a uterine manipulator, 3) minimal handling of the uterine cervix, and 4) bagging of the specimen (57).

Overall, the leading strength of this study was that it compared the efficacy of the ORH and LRH for the early cervical cancer patients comprising the new FIGO staging criterion. Then the study adopted the IPTW analysis to balance the prognostic risk factors between the two groups, even for the subgroup analysis. However, there are still several limitations of our study. In essence, this is a retrospectively single-center analysis. Moreover, variation of the surgeon’s operative experience on the rates of OS and PFS was not explored. Besides, since the robotic surgery was not adopted in our institution, we did not include patients accepting robotic radically hysterectomy. A recent meta-analysis conducted by Shazly et al. identified that laparoscopy and robotic RH are equivalent in terms of perioperative outcomes (5, 58). Furthermore, Gallotta et al. discovered that robotic RH and LRH had comparable perioperative, postoperative and survival outcomes for early cervical cancer patients through a large case matched control study. Therefore, robotic RH might also not play better roles than ORH for early stage cervical cancer. But to better identify the role of robotic RH, an ongoing prospective, international, multi-institutional, open-label randomized controlled Robot-assisted Approach to Cervical Cancer (RACC) trial is performed (15). It is worth mentioning that manipulator is prohibited, and patients with tumor size more than 4 cm were excluded in that study. So the research results were very worth expecting. Of course, none of these concerns alter the results of our primary outcome of interest.

In conclusion, this study confirms the superiority of open surgery on overall and progression free survival for early cervical cancer patients, no matter under FIGO 2009 or FIGO 2018 staging system. However, in subset of FIGO 2018 IA2, IB1-IB2, and IIA1, laparoscopic surgery showed non-inferiority, especially in patients with no prognostic risks. Considering the advantages and popularity of the minimally invasive surgery, laparoscopic radically hysterectomy should not be completely prohibited in early cervical cancer patients. In brief, ORH was recommended for early stage cervical cancer patients under 2018 FIGO staging system. But LRH might be suitable for early-stage cervical cancer patients without high and intermediate risk factors, including negative nodes, negative margins, negative parametria, less than 1/3 stromal invasion, negative LVSI and tumor size less than 2 cm.

## Data Availability Statement

The raw data supporting the conclusions of this article will be made available by the authors, without undue reservation.

## Ethics Statement

Written informed consent was obtained from the individual(s) for the publication of any potentially identifiable images or data included in this article.

## Author Contributions

QY and FB designed and edited this study. YX and WZ enrolled and followed up the patients. WCZ analyzed the data and wrote the manuscript. All authors contributed to the article and approved the submitted version.

## Funding

This work was supported by the National Natural Science Foundation of China (No. 81872125). This work was also supported by grants from 345 Talent Project of Shengjing hospital of China medical university (No. M0695).

## Conflict of Interest

The authors declare that the research was conducted in the absence of any commercial or financial relationships that could be construed as a potential conflict of interest.

## Supplementary Material

Supplementary Figure 1 Survival and recurrence outcomes between open and laparoscopic radically hysterectomy for stage IIIC1-IIIC2 cervical cancer patients based on Federation International of Gynecology and Obstetrics (FIGO) 2018 staging system. **(A)** Overall survival (OS) curves of the patients before propensity score-based inverse probability of treatment weighting (PS-IPTW) analysis. **(B)** OS curves of the patients after IPTW analysis. **(C)** Progression free survival (PFS) curves of the patients before IPTW analysis. **(D)** PFS curves of the patients after IPTW analysis.

Supplementary Figure 2 Survival and recurrence outcomes between open and laparoscopic radically hysterectomy for stage IA2, IB1, IB2 and IIA1 cervical cancer patients based on Federation International of Gynecology and Obstetrics (FIGO) 2018 staging system. **(A)** Overall survival (OS) curves of the patients before propensity score-based inverse probability of treatment weighting (PS-IPTW) analysis. **(B)** OS curves of the patients after IPTW analysis. **(C)** Progression free survival (PFS) curves of the patients before IPTW analysis. **(D)** PFS curves of the patients after IPTW analysis.

Supplementary Figure 3 Survival and recurrence outcomes between open and laparoscopic radically hysterectomy for stage IA2, IB1, IB2 and IIA1cervical cancer patients with no high and intermediate risks based on Federation International of Gynecology and Obstetrics (FIGO) 2018 staging system. **(A)** Overall survival (OS) curves of the patients before propensity score-based inverse probability of treatment weighting (PS-IPTW) analysis. **(B)** OS curves of the patients after IPTW analysis. **(C)** Progression free survival (PFS) curves of the patients before IPTW analysis. **(D)** PFS curves of the patients after IPTW analysis.

Supplementary Figure 4 Survival and recurrence outcomes between open and laparoscopic radically hysterectomy for stage IB3 and IIA2 cervical cancer patients based on Federation International of Gynecology and Obstetrics (FIGO) 2018 staging system. **(A)** Overall survival (OS) curves of the patients before propensity score-based inverse probability of treatment weighting (PS-IPTW) analysis. **(B)** OS curves of the patients after IPTW analysis. **(C)** Progression free survival (PFS) curves of the patients before IPTW analysis. **(D)** PFS curves of the patients after IPTW analysis.

## References

[1] Arbyn M Weiderpass E Bruni L de Sanjosé S Saraiya M Ferlay J. Estimates of Incidence and Mortality of Cervical Cancer in 2018: A Worldwide Analysis. Lancet Global Health (2020) 8:e191–203. 10.1016/S2214-109X(19)30482-6 PMC7025157 31812369

[2] Nezhat CR Burrell MO Nezhat FR Benigno BB Welander CE. Laparoscopic Radical Hysterectomy With Paraaortic and Pelvic Node Dissection. Am J Obstet Gynecol (1992) 166(3):864–65. 10.1016/0002-9378(92)91351-A 1532291

[3] Uppal S Rebecca Liu J Kevin Reynolds R Rice LW Spencer RJ. Trends and Comparative Effectiveness of Inpatient Radical Hysterectomy for Cervical Cancer in the United States (2012-2015). Gynecol Oncol (2019) 152:133–8. 10.1016/j.ygyno.2018.09.027 30424895

[4] Cao T Feng Y Huang Q Wan T Liu J. Prognostic and Safety Roles in Laparoscopic *Versus* Abdominal Radical Hysterectomy in Cervical Cancer: A Meta-Analysis. J Laparoendosc Adv Surg Tech A (2015) 25(12):990–998. 10.1089/lap.2015.0390 26584414 PMC4691653

[5] Shazly SA Murad MH Dowdy SC Gostout BS Famuyide AO. Robotic Radical Hysterectomy in Early Stage Cervical Cancer: A Systematic Review and Meta-Analysis. Gynecol Oncol (2015) 138:457–71. 10.1016/j.ygyno.2015.06.009 26056752

[6] Nitecki R Ramirez PT Frumovitz M Krause KJ Tergas AI Wright JD. Survival After Minimally Invasive *vs* Open Radical Hysterectomy for Early-Stage Cervical Cancer: A Systematic Review and Meta-Analysis. JAMA Oncol (2020) 6:1019–27. 10.1001/jamaoncol.2020.1694 PMC7290695 32525511

[7] Ramirez PT Frumovitz M Pareja R Lopez A Vieira M Ribeiro R. Minimally Invasive *Versus* Abdominal Radical Hysterectomy for Cervical Cancer. N Engl J Med (2018) 379:1895–904. 10.1056/NEJMoa1806395 30380365

[8] Diver E Hinchcliff E Gockley A Melamed A Contrino L Feldman S. Implementation of Laparoscopic Approach for Type B Radical Hysterectomy: A Comparison With Open Surgical Operations. Eur J Surg Oncol (2015) 41:34–9. 10.1016/j.ejso.2014.10.058 25468458

[9] Melamed A Margul DJ Chen L Keating NL Del Carmen MG Yang J. Survival After Minimally Invasive Radical Hysterectomy for Early-Stage Cervical Cancer. N Engl J Med (2018) 379:1905–14. 10.1056/NEJMoa1804923 PMC6464372 30379613

[10] Gallotta V Conte C Federico A Vizzielli G Gueli Alletti S Tortorella L. Robotic *Versus* Laparoscopic Radical Hysterectomy in Early Cervical Cancer: A Case Matched Control Study. Eur J Surg Oncol (2018) 44:754–9. 10.1016/j.ejso.2018.01.092 29422253

[11] Corrado G Vizza E Legge F Pedone Anchora L Sperduti I Fagotti A. Comparison of Different Surgical Approaches for Stage Ib1 Cervical Cancer Patients: A Multi-Institution Study and a Review of the Literature. Int J Gynecol Cancer (2018) 28:1020–8. 10.1097/IGC.0000000000001254 29727351

[12] Nam JH Park JY Kim DY Kim JH Kim YM Kim YT. Laparoscopic *Versus* Open Radical Hysterectomy in Early-Stage Cervical Cancer: Long-Term Survival Outcomes in a Matched Cohort Study. Ann Oncol (2012) 23:903–11. 10.1093/annonc/mdr360 21841155

[13] Park JY Kim DY Kim JH Kim YM Kim YT Nam JH. Laparoscopic Compared With Open Radical Hysterectomy in Obese Women With Early-Stage Cervical Cancer. Obstet Gynecol (2012) 119:1201–9. 10.1097/AOG.0b013e318256ccc5 22617585

[14] Diver E Hinchcliff E Gockley A Melamed A Contrino L Feldman S. Minimally Invasive Radical Hysterectomy for Cervical Cancer Is Associated With Reduced Morbidity and Similar Survival Outcomes Compared With Laparotomy. J Minimal Invasive Gynecol (2017) 24:402–6. 10.1016/j.jmig.2016.12.005 28011096

[15] Chiantera V Vizzielli G Lucidi A Gallotta V Petrillo M Legge F. Laparoscopic Radical Hysterectomy in Cervical Cancer as Total Mesometrial Resection (L-TMMR): A Multicentric Experience. Gynecol Oncol (2015) 139:47–51. 10.1016/j.ygyno.2015.07.010 26166805

[16] Gallotta V Conte C D’Indinosante M Federico A Biscione A Vizzielli G. Robotic Surgery in Elderly and Very Elderly Gynecologic Cancer Patients. J Minim Invasive Gynecol (2018) 25:872–7. 10.1016/j.jmig.2018.01.007 29339300

[17] Park DA Yun JE Kim SW Lee SH. Surgical and Clinical Safety and Effectiveness of Robot-Assisted Laparoscopic Hysterectomy Compared to Conventional Laparoscopy and Laparotomy for Cervical Cancer: A Systematic Review and Meta-Analysis. Eur J Surg Oncol (2017) 43:994–1002. 10.1016/j.ejso.2016.07.017 27546015

[18] Wang Y Li B Ren F Song Z Ouyang L Liu K. Survival After Minimally Invasive *vs*. Open Radical Hysterectomy for Cervical Cancer: A Meta-Analysis. Front Oncol (2020) 10:1236. 10.3389/fonc.2020.01236 32903313 PMC7396529

[19] Chiva L Zanagnolo V Querleu D Martin-Calvo N Arevalo-Serrano J Capilna ME. SUCCOR Study: An International European Cohort Observational Study Comparing Minimally Invasive Surgery *Versus* Open Abdominal Radical Hysterectomy in Patients With Stage IB1 Cervical Cancer. Int J Gynecol Cancer (2020) 30:1269–77. 10.1136/ijgc-2020-001506 32788262

[20] Margul DJ Yang J Seagle BL Kocherginsky M Shahabi S. Outcomes and Costs of Open, Robotic, and Laparoscopic Radical Hysterectomy for Stage IB1 Cervical Cancer. J Clin Oncol (2018) 36:5502–2. 10.1200/JCO.2018.36.15_suppl.5502

[21] Borcoman E Le Tourneau C. Pembrolizumab in Cervical Cancer: Latest Evidence and Clinical Usefulness. Ther Adv Med Oncol (2017) 9:431–9. 10.1177/1758834017708742 PMC5455883 28607581

[22] Ryu SY Kim MH Nam BH Lee TS Song ES Park CY. Intermediate-Risk Grouping of Cervical Cancer Patients Treated With Radical Hysterectomy: A Korean Gynecologic Oncology Group Study. Br J Cancer (2014) 110:278–85. 10.1038/bjc.2013.716 PMC3899760 24357798

[23] Querleu D Morrow CP. Classification of Radical Hysterectomy. Gynecol Oncol (2009) 115(2):314e5. 10.1016/j.ygyno.2009.07.027 19695686

[24] Mantel N. Evaluation of Survival Data and Two New Rank Order Statistics Arising in its Consideration. Cancer Chemother Rep (1966) 50(3):163–70. 5910392

[25] Kaplan EL Meier P. Nonparametric Estimation From Incomplete Samples. J Am Stat Assoc (1958) 53:457–81. No 282.

[26] Cox DR. Models and Life-Tables Regression. J R Stat Soc Ser B (Methodol) (1972) 34:187–220. No 2. 10.1111/j.2517-6161.1972.tb00899.x

[27] Lee J Little TD. A Practical Guide to Propensity Score Analysis for Applied Clinical Research. Behav Res Ther (2017) 98:76–90. 10.1016/j.brat.2017.01.005 28153337

[28] Bogani G Cromi A Uccella S Serati M Casarin J Pinelli C. Laparoscopic *Versus* Open Abdominal Management of Cervical Cancer: Long-Term Results From a Propensity-Matched Analysis. J Minim Invasive Gynecol (2014) 21(5):857–62. 10.1016/j.jmig.2014.03.018 24699300

[29] Shah CA Beck T Liao JB Giannakopoulos NV Veljovich D Paley P. Surgical and Oncologic Outcomes After Robotic Radical Hysterectomy as Compared to Open Radical Hysterectomy in the Treatment of Early Cervical Cancer. J Gynecol Oncol (2017) 28:e82. 10.3802/jgo.2017.28.e82 29027400 PMC5641532

[30] Alfonzo E Wallin E Ekdahl L Staf C Radestad AF Reynisson P. No Survival Difference Between Robotic and Open Radical Hysterectomy for Women With Early-Stage Cervical Cancer: Results From a Nationwide Population-Based Cohort Study. Eur J Cancer (2019) 116:169–77. 10.1016/j.ejca.2019.05.016 31200323

[31] Paik ES Lim MC Kim MH Kim YH Song ES Seong SJ. Comparison of Laparoscopic and Abdominal Radical Hysterectomy in Early Stage Cervical Cancer Patients Without Adjuvant Treatment: Ancillary Analysis of a Korean Gynecologic Oncology Group Study (Kgog 1028). Gynecol Oncol (2019) 154:547–53. 10.1016/j.ygyno.2019.06.023 31272738

[32] Yuan Z Cao D Yang J Yu M Shen K Yang J. Laparoscopic *vs*. Open Abdominal Radical Hysterectomy for Cervical Cancer: A Single-Institution, Propensity Score Matching Study in China. Front Oncol (2019) 9:1107. 10.3389/fonc.2019.01107 31737563 PMC6833183

[33] Mendivil AA Rettenmaier MA Abaid LN Brown JV 3rd Micha JP Lopez KL. Survival Rate Comparisons Amongst Cervical Cancer Patients Treated With an Open, Robotic-Assisted or Laparoscopic Radical Hysterectomy: A Five Year Experience. Surg Oncol (2016) 25:66–71. 10.1016/j.suronc.2015.09.004 26409687

[34] Bhatla N Berek JS Cuello Fredes M Denny LA Grenman S Karunaratne K. Revised FIGO Staging for Carcinoma of the Cervix Uteri. Int J Gynaecol Obstet (2019) 145:129–35. 10.1002/ijgo.12749 30656645

[35] Yang J Mead-Harvey C Polen-De C Magtibay P Butler K Cliby W. Survival Outcomes in Patients With Cervical Cancer Treated With Open *Versus* Robotic Radical Hysterectomy: Our Surgical Pathology Interrogation. Gynecol Oncol (2020) 159:373–80. 10.1016/j.ygyno.2020.08.031 32893029

[36] Sedlis A Bundy BN Rotman MZ Muderspach LI Zaino RJ. A Randomized Trial of Pelvic Radiation Therapy *Versus* No Further Therapy in Selected Patients With Stage IB Carcinoma of the Cervix After Radical Hysterectomy and Pelvic Lymphadenectomy: A Gynecologic Oncology Group Study. Gynecol Oncol (1999) 73:177–83. 10.1006/gyno.1999.5387 10329031

[37] Delgado G Bundy B Zaino R Sevin BU Creasman WT Major F. Prospective Surgical-Pathological Study of Disease-Free Interval in Patients With Stage IB Squamous Cell Carcinoma of the Cervix: A Gynecologic Oncology Group Study. Gynecol Oncol (1990) 38:352–7. 10.1016/0090-8258(90)90072-S 2227547

[38] Uppal S Gehrig PA Peng K Bixel KL Matsuo K Vetter MH. Recurrence Rates in Patients With Cervical Cancer Treated With Abdominal *Versus* Minimally Invasive Radical Hysterectomy: A Multi-Institutional Retrospective Review Study. J Clin Oncol (2020) 38:1030–40. 10.1200/JCO.19.03012 32031867

[39] Pedone Anchora L Turco LC Bizzarri N Capozzi VA Lombisani A Chiantera V. How to Select Early-Stage Cervical Cancer Patients Still Suitable for Laparoscopic Radical Hysterectomy: A Propensity-Matched Study. Ann Surg Oncol (2020) 27:1947–55. 10.1245/s10434-019-08162-5 31898100

[40] Li P Chen L Ni Y Liu J Li D Guo J. Comparison Between Laparoscopic and Abdominal Radical Hysterectomy for Stage IB1 and Tumor Size <2 Cm Cervical Cancer With Visible or Invisible Tumors: A Multicentre Retrospective Study. J Gynecol Oncol (2021) 32:e17. 10.3802/jgo.2021.32.e17 33470062 PMC7930457

[41] Fagotti A Pedone Anchora L Conte C Chiantera V Vizza E Tortorella L. Beyond Sentinel Node Algorithm. Toward a More Tailored Surgery for Cervical Cancer Patients. Cancer Med (2016) 5:1725–30. 10.1002/cam4.722 PMC4971900 27230108

[42] Ramirez PT Pareja R Rendon GJ Millan C Frumovitz M Schmeler KM. Management of Low-Risk Early-Stage Cervical Cancer: Should Conization, Simple Trachelectomy, or Simple Hysterectomy Replace Radical Surgery as the New Standard of Care? Gynecol Oncol (2014) 132:254–9. 10.1016/j.ygyno.2013.09.004 PMC4286394 24041877

[43] Wagner AE Pappas L Ghia AJ Gaffney DK. Impact of Tumor Size on Survival in Cancer of the Cervix and Validation of Stage IIA1 and IIA2 Subdivisions. Gynecol Oncol (2013) 129:517–21. 10.1016/j.ygyno.2013.03.008 23528928

[44] Noh JM Park W Kim YS Kim JY Kim HJ Kim J. Comparison of Clinical Outcomes of Adenocarcinoma and Adenosquamous Carcinoma in Uterine Cervical Cancer Patients Receiving Surgical Resection Followed by Radiotherapy: A Multicenter Retrospective Study (KROG 13-10). Gynecol Oncol (2014) 132:618–23. 10.1016/j.ygyno.2014.01.043 24486605

[45] Krizova A Clarke BA Bernardini MQ James S Kalloger SE Boerner SL. Histologic Artifacts in Abdominal, Vaginal, Laparoscopic, and Robotic Hysterectomy Specimens: A Blinded, Retrospective Review. Am J Surg Pathol (2011) 35:115–26. 10.1097/PAS.0b013e31820273dc 21164295

[46] Pedone Anchora L Carbone V Gallotta V Fanfani F Cosentino F Turco LC. Should the Number of Metastatic Pelvic Lymph Nodes be Integrated Into the 2018 Figo Staging Classification of Early Stage Cervical Cancer? Cancers (Basel) (2020) 12:1552. 10.3390/cancers12061552 PMC7352475 32545508

[47] Lin F Pan L Li L Li D Mo L. Effects of a Simulated CO2 Pneumoperitoneum Environment on the Proliferation, Apoptosis, and Metastasis of Cervical Cancer Cells *In Vitro* . Med Sci Monit (2014) 20:2497–503. 10.12659/MSM.891179 PMC4260668 25436974

[48] Volz J Köster S Spacek Z Paweletz N. The Influence of Pneumoperitoneum Used in Laparoscopic Surgery on an Intraabdominal Tumor Growth. Cancer (1999) 86:770–4. 10.1002/(SICI)1097-0142(19990901)86:5<770::AID-CNCR11>3.0.CO;2-3 10463974

[49] Kong TW Chang SJ Piao X Paek J Lee Y Lee EJ. Patterns of Recurrence and Survival After Abdominal *Versus* Laparoscopic/ Robotic Radical Hysterectomy in Patients With Early Cervical Cancer. J Obstet Gynaecol Res (2016) 42(1):77–86. 10.1111/jog.12840 26554751

[50] Lim S Kim HS Lee KB Yoo CW Park SY Seo SS. Does the Use of a Uterine Manipulator With an Intrauterine Balloon in Total Laparoscopic Hysterectomy Facilitate Tumor Cell Spillage Into the Peritoneal Cavity in Patients With Endometrial Cancer? Int J Gynecol Cancer (2008) 18:1145–9. 10.1111/j.1525-1438.2007.01165.x 18217979

[51] Rakowski JA Tran TA Ahmad S James JA Brudie LA Pernicon PJ. Does a Uterine Manipulator Affect Cervical Cancer Pathology or Identification of Lymphovascular Space Involvement? Gynecol Oncol (2012) 127:98–101. 10.1016/j.ygyno.2012.07.094 22800652

[52] Kohler C Hertel H Herrmann J Marnitz S Mallmann P Favero G. Laparoscopic Radical Hysterectomy With Transvaginal Closure of Vaginal Cuff - a Multicenter Analysis. Int J Gynecol Cancer (2019) 29:845–50. 10.1136/ijgc-2019-000388 31155516

[53] Uppal S Spencer R. Modify or Abandon: Minimally Invasive Radical Hysterectomy for Early-Stage Cervical Cancer. Int J Gynecol Cancer (2019) 29:843–4. 10.1136/ijgc-2019-000574 31155515

[54] Casarin J Buda A Bogani G Fanfani F Papadia A Ceccaroni M. Predictors of Recurrence Following Laparoscopic Radical Hysterectomy for Early-Stage Cervical Cancer: A Multi-Institutional Study. Gynecol Oncol (2020) 159:164–70. 10.1016/j.ygyno.2020.06.508 32665147

[55] Klapdor R Hertel H Hillemanns P Rottger M Soergel P Kuehnle E. Peritoneal Contamination With ICG-stained Cervical Secretion as Surrogate for Potential Cervical Cancer Tumor Cell Dissemination: A Proof-of-Principle Study for Laparoscopic Hysterectomy. Acta Obstet Gynecol Scand (2019) 98:1398–403. 10.1111/aogs.13681 31242322

[56] Pedone Anchora L Bizzarri N Kucukmetin A Turco LC Gallotta V Carbone V. Investigating the Possible Impact of Peritoneal Tumor Exposure Amongst Women With Early Stage Cervical Cancer Treated With Minimally Invasive Approach. Eur J Surg Oncol (2021) 47:1090–7. 10.1016/j.ejso.2020.09.038 33039294

[57] Kanao H Matsuo K Aoki Y Tanigawa T Nomura H Okamoto S. Feasibility and Outcome of Total Laparoscopic Radical Hysterectomy With No-Look No-Touch Technique for FIGO IB1 Cervical Cancer. J Gynecol Oncol (2019) 30:e71. 10.3802/jgo.2019.30.e71 30887768 PMC6424854

[58] Gallotta V Conte C Federico A Vizzielli G Gueli Alletti S Tortorella L. Robotic *Versus* Laparoscopic Radical Hysterectomy in Early Cervical Cancer: A Case Matched Control Study. Eur J Surg Oncol (2018) 44:754–9. 10.1016/j.ejso.2018.01.092 29422253

