# Supplementary figures and images for: Survival Outcomes in Patients With 2018 FIGO Stage IA2–IIA2 Cervical Cancer Treated With Laparoscopic Versus Open Radical Hysterectomy: A Propensity Score-Weighting Analysis
Source: Front Oncol. 2021 Jun 17;11:682849. doi: 10.3389/fonc.2021.682849 (PMC8247576; doi:10.3389/fonc.2021.682849)

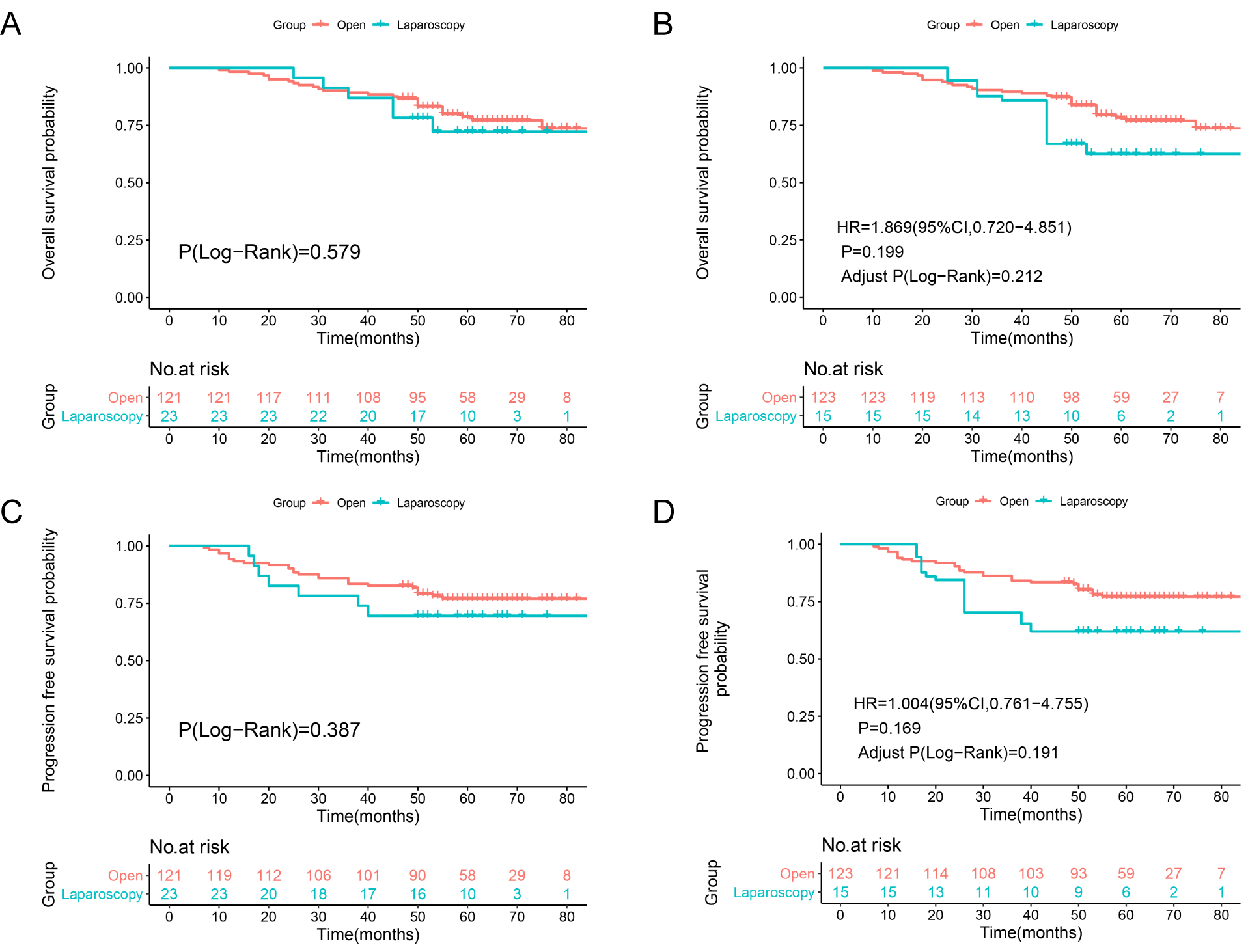

Supplement: Supplementary Figure 1 — Survival and recurrence outcomes between open and laparoscopic radically hysterectomy for stage IIIC1-IIIC2 cervical cancer patients based on Federation International of Gynecology and Obstetrics (FIGO) 2018 staging system. (A) Overall survival (OS) curves of the patients before propensity score-based inverse probability of treatment weighting (PS-IPTW) analysis. (B) OS curves of the patients after IPTW analysis. (C) Progression free survival (PFS) curves of the patients before IPTW analysis. (D) PFS curves of the patients after IPTW analysis. [file Image_1.tif]

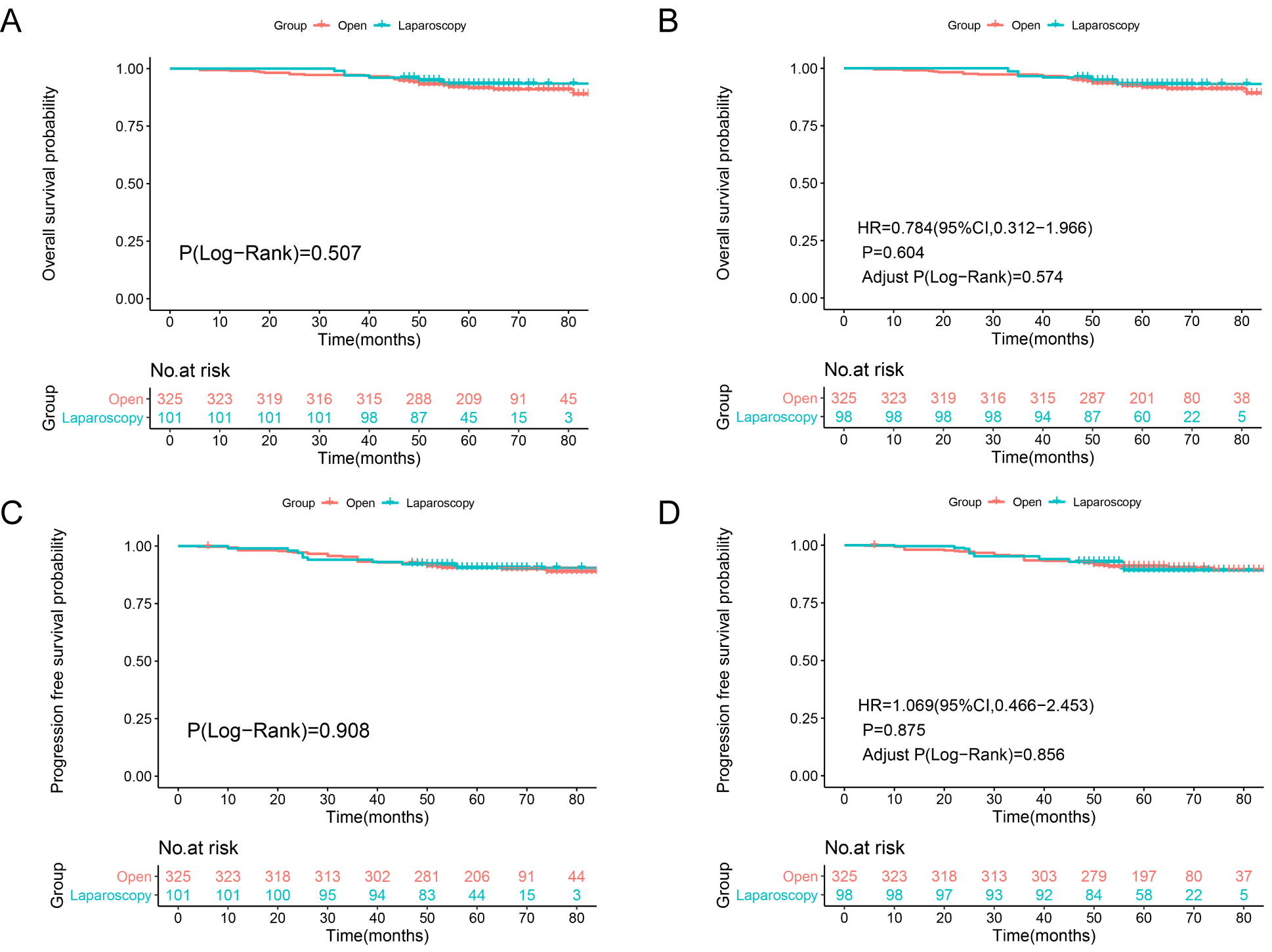

Supplement: Supplementary Figure 2 — Survival and recurrence outcomes between open and laparoscopic radically hysterectomy for stage IA2, IB1, IB2 and IIA1 cervical cancer patients based on Federation International of Gynecology and Obstetrics (FIGO) 2018 staging system. (A) Overall survival (OS) curves of the patients before propensity score-based inverse probability of treatment weighting (PS-IPTW) analysis. (B) OS curves of the patients after IPTW analysis. (C) Progression free survival (PFS) curves of the patients before IPTW analysis. (D) PFS curves of the patients after IPTW analysis. [file Image_2.tif]

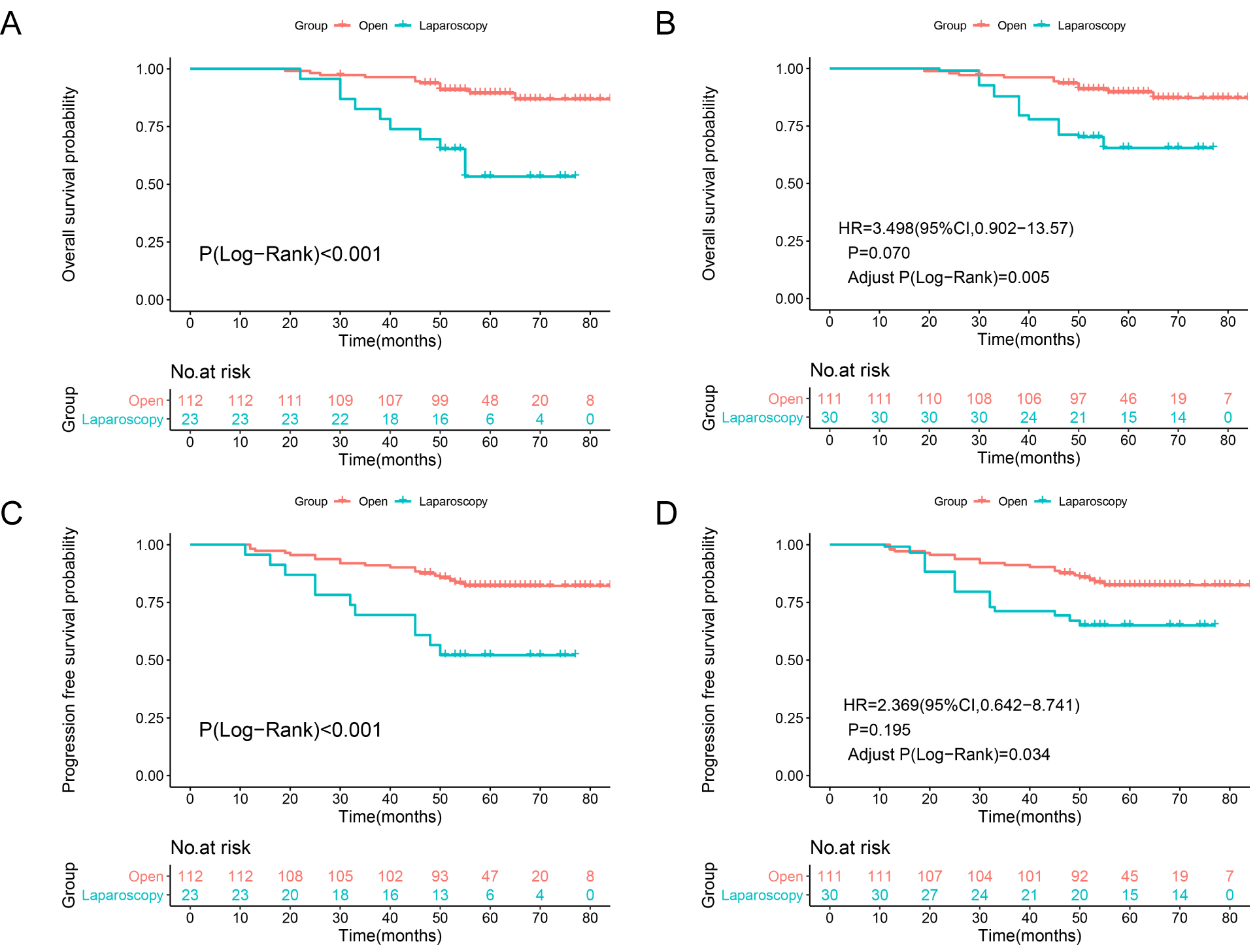

Supplement: Supplementary Figure 3 — Survival and recurrence outcomes between open and laparoscopic radically hysterectomy for stage IA2, IB1, IB2 and IIA1cervical cancer patients with no high and intermediate risks based on Federation International of Gynecology and Obstetrics (FIGO) 2018 staging system. (A) Overall survival (OS) curves of the patients before propensity score-based inverse probability of treatment weighting (PS-IPTW) analysis. (B) OS curves of the patients after IPTW analysis. (C) Progression free survival (PFS) curves of the patients before IPTW analysis. (D) PFS curves of the patients after IPTW analysis. [file Image_3.tif]

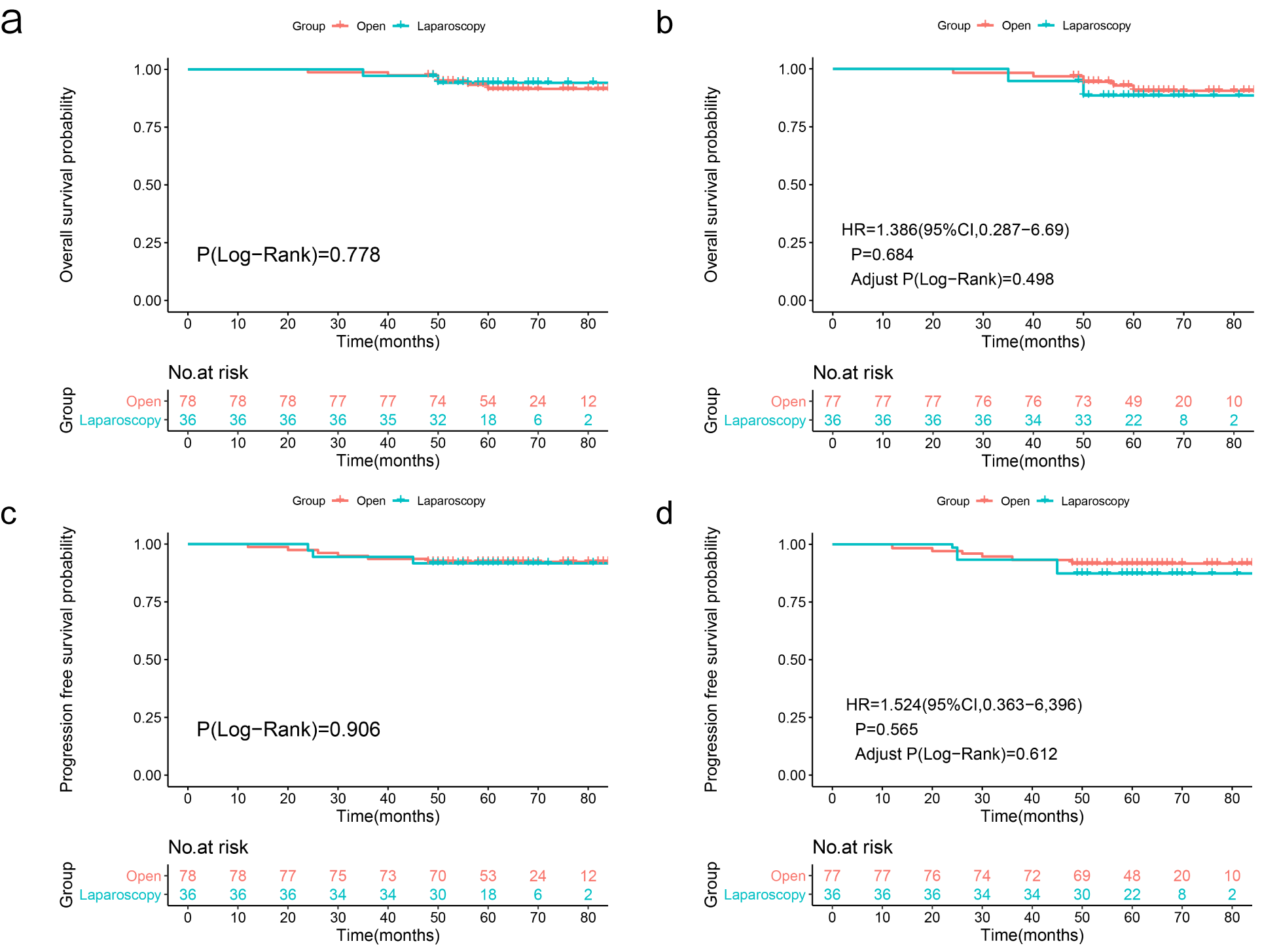

Supplement: Supplementary Figure 4 — Survival and recurrence outcomes between open and laparoscopic radically hysterectomy for stage IB3 and IIA2 cervical cancer patients based on Federation International of Gynecology and Obstetrics (FIGO) 2018 staging system. (A) Overall survival (OS) curves of the patients before propensity score-based inverse probability of treatment weighting (PS-IPTW) analysis. (B) OS curves of the patients after IPTW analysis. (C) Progression free survival (PFS) curves of the patients before IPTW analysis. (D) PFS curves of the patients after IPTW analysis. [file Image_4.tif]
