# Supplementary material for: Survival Outcomes in Patients With 2018 FIGO Stage IA2–IIA2 Cervical Cancer Treated With Laparoscopic Versus Open Radical Hysterectomy: A Propensity Score-Weighting Analysis
Source: Front Oncol. 2021 Jun 17;11:682849. doi: 10.3389/fonc.2021.682849 (PMC8247576; doi:10.3389/fonc.2021.682849)
Supplement: Supplementary file 5 [file Table_1.docx]

Table 1

Distribution of patients’ clinical stages according to FIGO 2009 and FIGO 2018 criteria respectively.

| **FIGO 2009**  **(n=705)** | **FIGO 2018 (n=705)** | | | | | | | |
| --- | --- | --- | --- | --- | --- | --- | --- | --- |
|  | **IA2** | **IB1** | **IB2** | **IB3** | **IIA1** | **IIA2** | **IIIC1p** | **IIIC2p** |
| **IA2** | 13(1.8) | － | － | － | － | － | － | － |
| **IB1** | － | 82(11.6) | 223(31.6) | 9(12.8) | － | － | 56(7.9) | 2(0.3) |
| **IB2** | － | － | － | 54(7.7) | － | － | 16(2.3) | 1(0.1) |
| **IIA1** | － | － | － | － | 108(15.3) | － | 40(5.7) | 3(0.4) |
| **IIA2** | － | － | － | － | － | 72(10.2) | 26(3.7) | － |

Abbreviations: FIGO,Federation International of Gynecology and Obstetrics.p, pathology.
